# Vaccination with live attenuated simian immunodeficiency virus causes dynamic changes in intestinal CD4+CCR5+ T cells

**DOI:** 10.1186/1742-4690-8-8

**Published:** 2011-02-03

**Authors:** Bo Li, Neil Berry, Claire Ham, Deborah Ferguson, Deborah Smith, Joanna Hall, Mark Page, Ruby Quartey-Papafio, William Elsley, Mark Robinson, Neil Almond, Richard Stebbings

**Affiliations:** 1 Biotherapeutics Group, National Institute of Biological Standards and Control/Health Protection Agency, Potters Bar, Hertfordshire, UK; 2 Division of Retrovirology, National Institute of Biological Standards and Control/Health Protection Agency, Potters Bar, Hertfordshire, UK

## Abstract

**Background:**

Vaccination with live attenuated SIV can protect against detectable infection with wild-type virus. We have investigated whether target cell depletion contributes to the protection observed. Following vaccination with live attenuated SIV the frequency of intestinal CD4+CCR5+ T cells, an early target of wild-type SIV infection and destruction, was determined at days 3, 7, 10, 21 and 125 post inoculation.

**Results:**

In naive controls, modest frequencies of intestinal CD4+CCR5+ T cells were predominantly found within the LPL T_TrM-1 _and IEL T_TrM-2 _subsets. At day 3, LPL and IEL CD4+CCR5+ T_EM _cells were dramatically increased whilst less differentiated subsets were greatly reduced, consistent with activation-induced maturation. CCR5 expression remained high at day 7, although there was a shift in subset balance from CD4+CCR5+ T_EM _to less differentiated T_TrM-2 _cells. This increase in intestinal CD4+CCR5+ T cells preceded the peak of SIV RNA plasma loads measured at day 10. Greater than 65.9% depletion of intestinal CD4+CCR5+ T cells followed at day 10, but overall CD4+ T cell homeostasis was maintained by increased CD4+CCR5- T cells. At days 21 and 125, high numbers of intestinal CD4+CCR5- naive T_N _cells were detected concurrent with greatly increased CD4+CCR5+ LPL T_TrM-2 _and IEL T_EM _cells at day 125, yet SIV RNA plasma loads remained low.

**Conclusions:**

This increase in intestinal CD4+CCR5+ T cells, following vaccination with live attenuated SIV, does not correlate with target cell depletion as a mechanism of protection. Instead, increased intestinal CD4+CCR5+ T cells may correlate with or contribute to the protection conferred by vaccination with live attenuated SIV.

## Background

Non-human primates (NHP) challenged with simian immunodeficiency virus (SIV) or engineered SIV/HIV-1 chimeras (SHIV) have been used as models to evaluate the efficacy of a wide variety of candidate AIDS vaccine approaches for more than two decades [1-6]. Amongst the vaccine strategies evaluated in NHP models, vaccination with live attenuated SIV/SHIV has proven to be the most effective at providing broad protective immunity against a wide range of SIV and SHIV challenges [7-15]. However, concerns regarding the safety of a live attenuated SIV or HIV vaccine have to date limited further pursuit of this approach as an AIDS vaccine strategy in the clinic [16-20]. Nevertheless, the potency of this vaccine protection has led to further studies in NHP models to provide information on the mechanisms of protective immunity that a safe and effective human vaccine may have to reproduce to be of equal efficacy [21].

Many groups have attempted to identify robust correlates of protection amongst the adaptive immune responses elicited by live attenuated SIV vaccines. Unfortunately a confusing picture has developed, with different groups reporting either partial, full or no correlation with various measures of adaptive immunity [22-39]. This confusion may have resulted from the range of different NHP models used for these studies: using different vaccines, different challenge viruses and different species of macaque. However, since the efficacy of live attenuated vaccines appears to correlate inversely with the level of attenuation [40,41] and the most effective vaccines persist and replicate in the host [42], then it is possible that innate responses may also contribute to the vaccine effect [36,37]. This would appear to be the case with live attenuated vaccines that have been reported to protect within as little as 3 weeks vaccination when adaptive antiviral immune responses are low or absent [43].

The gut-associated lymphoid tissue (GALT) constitutes a large immune compartment within the body [44-47] which, compared to other lymphoid compartments, is rich in CD4+ T cells expressing CCR5 [48-50], a preferential co-receptor for HIV and SIV infection [51-53]. Early depletion of intestinal CD4+CCR5+ T cells is now a recognised hallmark of wild-type SIV/HIV infection resulting from the destruction of virus infected target cells [46,48,54-57]. It could be hypothesised that if live attenuated SIV vaccines caused a similar loss of CD4+CCR5+ T cells in this compartment, then this depletion of target cells could contribute to the vaccine effect. However, it has been reported that vaccination of rhesus macaques with live attenuated SIV does not cause significant loss of intestinal CD4+ T cells [48,58]. Moreover, it has recently been reported that vaccination with attenuated SIV causes a transient increase in activated CD4+ memory T cells [58]. Nonetheless, dynamic changes in CCR5 expression within intestinal CD4+ T cell memory subsets were not assessed in detail, nor have these types of studies been performed in models involving cynomolgus macaques.

In the present study, we have characterised the impact on CD4+CCR5+ intestinal T cell memory subsets following inoculation with a potent live *nef*-attenuated SIV vaccine in the cynomolgus macaque model. These data have revealed that vaccination results in dramatic dynamic changes in key lymphocyte subsets in the intestinal tract that appear to be more consistent with immune activation, likely to induce innate and adaptive responses, than target cell depletion. These changes may contribute not only to the kinetics of vaccine protection, but also to the kinetics of virus replication.

## Results

### Attenuated SIV virus loads in blood and lymphoid tissues peak at day 10

**Figure 1:**
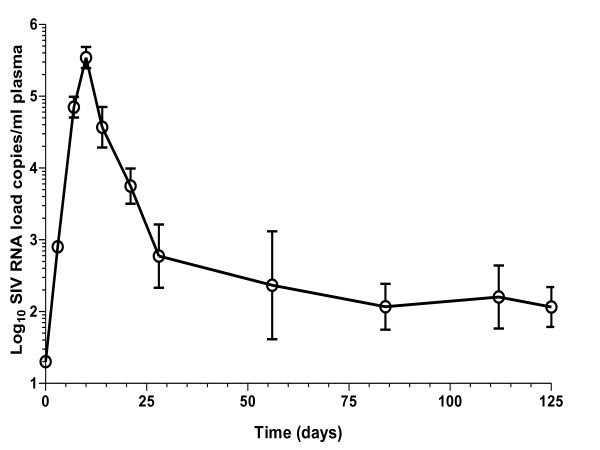
**Viral RNA dynamics in macaques vaccinated with attenuated SIV**. Following vaccination of cynomolgus macaques (n = 20) with live attenuated SIV plasma and lymphoid tissue viral loads was determined at days 0, 3, 7, 10, 21 and 125 post inoculation. Mean attenuated SIV RNA plasma levels peaked at day 10 with a nadir between days 84 and 125. For analysis n = 16 at day 3 reducing to n = 6 by day 21 as animals were sacrificed, n = 2 at all time points thereafter. Error bars shown are ± 1 SEM.

Following inoculation with live attenuated SIV, plasma SIV RNA loads (copies/ml) increased significantly at days 3 and 7 (log_10 _2.90 ± 0.08, p < 0.001 and log_10 _4.85 ± 0.14, p < 0.001 respectively) compared to naive controls, peaking at day 10 (log_10 _5.54 ± 0.15, p < 0.001; Figure 1). Compared with day 10, SIV RNA loads declined significantly by days 14 and 21 (log_10 _4.57 ± 0.28, p < 0.001 and log_10 _3.75 ± 0.25, p < 0.001, respectively) onwards to nadir between days 84 and 125 (log_10 _2.07 ± 0.32 and log_10 _2.06 ± 0.28, respectively; Figure 1). Mean levels of <1 SIV DNA copies per 100,000 small intestine (SI) lymphocytes measured at days 3 and 7 contrasted with peak loads at day 10 (105 ± 85), but were reduced thereafter at days 21 (21 ± 17) and 125 (2 ± 1). Cell-associated intestinal lymphocytes virus loads were measured at day 10 (log_10 _2.25 ± 0.75 SIV producing cells per 10^6 ^cells), but declined below detection limits by day 21 for all intestinal cell samples (data not shown).

### Attenuated SIV does not cause overt depletion of intestinal CD4+ T cells

**Figure 2:**
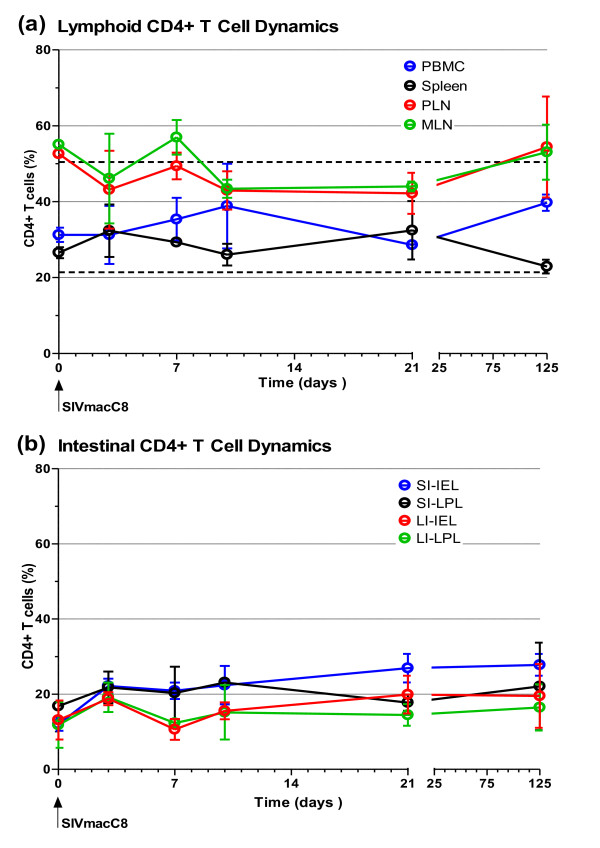
**CD4+ T cell dynamics in macaques vaccinated with attenuated SIV**. Following vaccination of cynomolgus macaques (n = 20) with live attenuated SIV peripheral blood, lymphoid tissue and intestinal lymphocyte CD4+ T cell percentages was determined at days 0, 3, 7, 10, 21 and 125 post inoculation. No evidence of overt CD4+ T cell depletion was detected in peripheral blood, lymphoid tissues **(a)**, or intestinal lamina propria and intraepithelial lymphocytes of the small and large intestine **(b)**. The mean range of CD4+ T cell percentages in peripheral blood derived from 335 naïve cynomolgus macaques ± 2 standard deviations is 35.9% ± 14.52, shown as a pair of black dashed lines. For analysis of peripheral blood n = 16 at day 3 reducing to n = 6 by day 21 as animals were sacrificed, n = 2 at all time points thereafter. For analysis of tissues n = 4 at all time points except days 7 and 125 where n = 2. Error bars shown are ± 1 SEM. PBMC: peripheral blood mononuclear cells, PLN: peripheral lymph node, MLN: mesenteric lymph node, SI: small intestine, LI: large intestine, IEL: intraepithelial lymphocytes, LPL: lamina propria lymphocytes.

Following vaccination with live attenuated SIV, no significant change in the total percentage of CD4+ T cells in peripheral blood mononuclear cells (PBMC), peripheral lymph node cells (PLN), mesenteric lymph node cells (MLN) or spleen was observed at days 3, 7, 10, 21 and 125 compared with naïve controls (Figure 2a). It was noted that percentages of CD4+ T cells in peripheral blood fluctuated following vaccination with live attenuated SIV but remained within normal reference range (Figure 2a). Detailed analysis of intra-epithelial lymphocytes (IEL) and lamina propria lymphocytes (LPL) from both the SI and large intestine (LI) did not reveal any significant changes in the total percentage of CD4+ T cells following vaccination with live attenuated SIV. A trend towards an increase in the percentage of CD4+ T cells over time was noted (Figure 2b). However, this trend was not significant.

### Attenuated SIV causes dynamic changes in intestinal CD4+CCR5+ T cells

**Figure 3:**
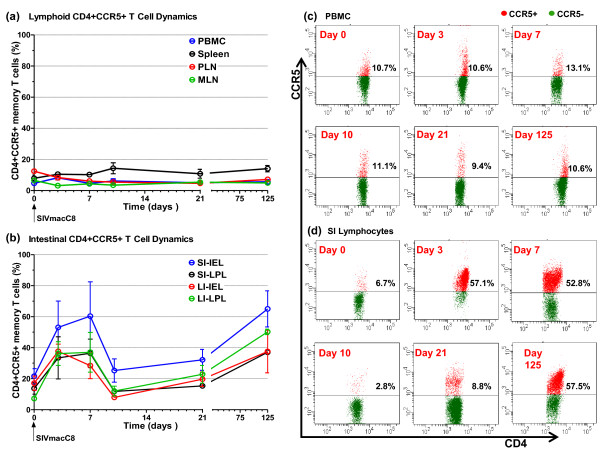
**CCR5+ T cell dynamics in macaques vaccinated with attenuated SIV**. Following vaccination of cynomolgus macaques (n = 20) with live attenuated SIV peripheral blood, lymphoid tissue and intestinal lymphocyte CD4+CCR5+ memory T cell percentages was determined at days 0, 3, 7, 10, 21 and 125 post inoculation. There was no evidence of dynamic changes in percentages of CD4+CCR5+ memory T cell in peripheral blood and lymphoid tissues **(a)**. In contrast, dynamic changes in CD4+CCR5+ memory T cell percentages was observed in the lamina propria and intraepithelial lymphocytes of both the small and large intestine **(b)**. Panels **(c) **and **(d) **shows representative staining for CCR5 on CD4+ PBMC and SI lymphocytes, respectively, at each time point. CCR5+CD4+ T cells are shown in red and CCR5-CD4+ T cells in green. For analysis of peripheral blood n = 16 at day 3 reducing to n = 6 by day 21 as animals were sacrificed, n = 2 at all time points thereafter. For analysis of tissues n = 4 at all time points except days 7 and 125 where n = 2. Error bars shown are ± 1 SEM. PBMC: peripheral blood mononuclear cells, PLN: peripheral lymph node, MLN: mesenteric lymph node, SI: small intestine, LI: large intestine, IEL: intraepithelial lymphocytes, LPL: lamina propria lymphocytes.

Analysis of CCR5 expression by CD4+ T cells focused on memory subsets since naive cells were predominantly CCR5 negative. No significant changes in the proportion of CD4+CCR5+ memory T cells within PBMC, PLN, MLN or spleen were observed following vaccination with live attenuated SIV (Figure 3a). By contrast, vaccination with live attenuated SIV resulted in a marked increase in the mean frequency of all 4 subpopulations of intestinal CD4+CCR5+ memory T cells taken together at days 3 (40.54% ± 6.24%, p <0.05) and 7 (40.54% ± 7.01%, p <0.05) compared with naive controls. The mean level of intestinal CD4+ T cells positive for CCR5 expression in naive controls was 16.20% ± 2.44%. Remarkably, at day 10 the frequency of intestinal CD4+CCR5+ memory T cells returned to levels observed in naive macaques (16.45% ± 3.71%, p = 0.9), apparently wiping out the earlier post vaccination expansion. At day 21, a small increase in intestinal CD4+CCR5+ memory T cells (22.50% ± 2.76%, p = 0.16) was not significant. However, the frequency of this cell population increased significantly at day 125 (47.46% ± 5.51%, p < 0.05), with the caveat that n = 2 at this time point. More detailed analysis of increased intestinal CD4+CCR5+ memory T cells at days 3, 7 and 125 revealed that these changes occurred in both the IEL and LPL of the SI and LI (Figure 3b). Representative dot plots showing CCR5 staining of CD4+ PBMC and SI lymphocytes at each time point are shown in Figures 3c and 3d, respectively.

**Figure 4:**
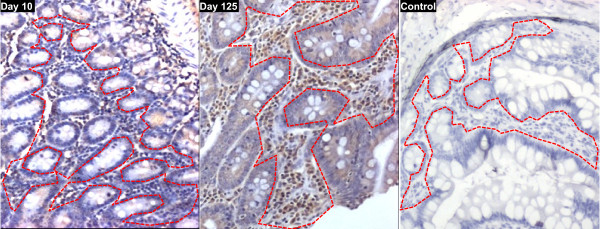
**Representative immunohistochemistry showing expression of CCR5 by lamina propria lymphocytes in the small intestine of macaques vaccinated with attenuated SIV**. Following vaccination of cynomolgus macaques (n = 20) with attenuated SIV immunohistochemical staining for CCR5+ cells was performed on sections of small intestine. At day 10 (left panel) a low frequency of CCR5+ cells (brown cell surface staining) was observed in the T cell areas of the lamina propria surrounding crypts (delineated by a dashed red line in each panel). In contrast, at day 125 (centre panel) a high frequency of CCR5+ cells was seen in the T cell areas of the lamina propria. The right hand panel shows a control slide with anti-CCR5 antibody omitted. Sections shown are from representative animals, counterstained with haematoxylin. Magnification is ×100.

Immunohistochemical analysis of CCR5 expression by LPL within SI sections of macaques vaccinated with attenuated SIV coincided with the early expansion of intestinal CD4+CCR5+ T cells seen by flow cytometry at the same time (data not shown). At day 10, a low frequency of CCR5+ LPL was observed by immunohistochemistry (Figure 4) which coincided with flow cytometry data showing depletion of intestinal CD4+CCR5+ T cells at that time. By immunohistochemistry the level of CCR5+ LPL at day 10 was similar to that of naive macaques (data not shown). In contrast, at day 125 the proportion of CCR5+ LPL revealed by immunohistochemistry was greatly increased (Figure 4), coinciding with expansion of intestinal CD4+CCR5+ T cells seen by flow cytometry at that time point.

### Attenuated SIV upregulates intestinal CD4+ T_CM _and T_EM _cell CCR5 expression

**Figure 5:**
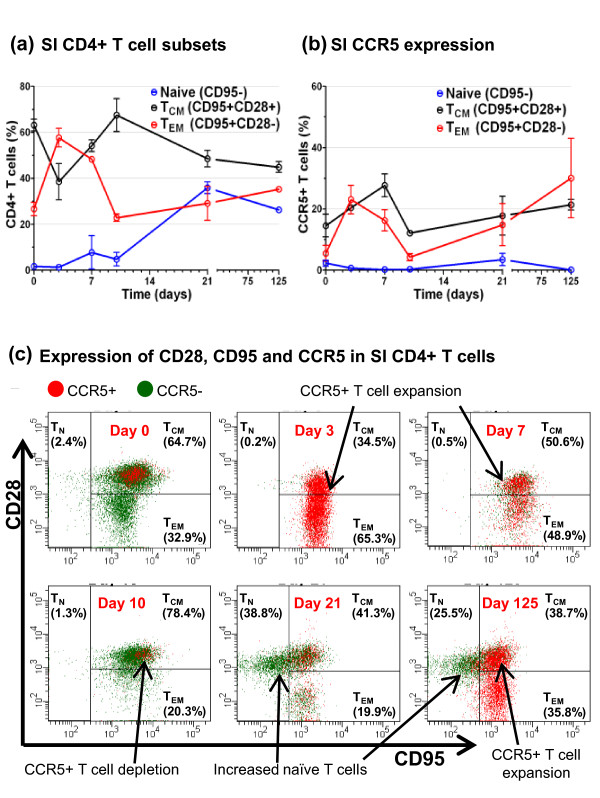
**CD4+CCR5+ T_CM _and T_EM _cell dynamics in the small intestine of macaques vaccinated with attenuated SIV**. Following vaccination of cynomolgus macaques (n = 20) with live attenuated SIV the percentages of CD4+CCR5+ T_CM _and T_EM _cells from the SI was determined at days 0, 3, 7, 10, 21 and 125 post inoculation. Dynamic changes in the frequency of SI CD4+ lymphocytes naïve, T_CM_, T_EM _subsets was observed following vaccination **(a)**. Increases in the percentage of CD4+CCR5+ T_CM _and T_EM _cells was observed at days 3 and 7, reversed at days 10 and 21, then increased again at day 125 compared to naive controls **(b)**. Immunostaining of SI lymphocytes gated on CD3+CD4+ T cells from one representative animal from each time point are shown **(c)**. In each panel the left hand quadrant shows CD28+CD95- T_N _cells, the upper right quadrant CD28+CD95+ T_CM _cells and the lower right quadrant CD28-CD95+ T_EM _cells. Percentages shown give the proportion present in each of these subsets. CD4+CCR5+ T cells are shown in red and CD4+CCR5- T cells in green. T_N_: naive, T_CM_: central memory, T_EM_: effector memory, SI: small intestine.

As in man, macaque CD4+ T cell populations can be subdivided into three distinct subpopulations: Naive (T_N_) which are quiescent and non-dividing, central memory (T_CM_) and effector memory (T_EM_) which are distinguished by the absence or presence of immediate effector function, respectively [59]. In cynomolgus macaques these CD4+ T cell subpopulations can be distinguished using a combination of anti-CD28 and anti-CD95 antibody markers [59,60]. Using these we found the intestinal CD4+T cells of naive cynomolgus macaques were almost entirely composed of CD95+CD28+ T_CM _and CD95+CD28- T_EM _memory cells, with relatively few, approximately 1%, CD95-CD28+ naive (T_N_) cells present (Figures 5a and 5c: day 0). Expression of CCR5 was confined primarily to a small fraction of CD4+ T_CM _cells, the T_EM _subset being largely CCR5- (Figures 5b and 5c: day 0). Following vaccination with live attenuated SIV, CCR5 expression was upregulated dramatically in both intestinal CD4+ T_CM _and T_EM _cells at days 3 (20.23% T_CM _± 1.84%, p < 0.05 and 23.03% T_EM _± 8.13%, p < 0.05) and 7 (23.96% T_CM _± 1.99%, p = 0.06 and 16.25% T_EM _± 6.09%, p = 0.08; Figures 5b and 5c). However, by day 10 CD4+CCR5+ T_CM _and T_EM _cells were reduced significantly compared with day 7 (11.74% T_CM _± 1.18%, p = 0.08 and 4.28% T_EM _± 1.13%, p = 0.08), the remaining intestinal CD4+ T cells being predominantly CCR5- T_CM _cells due to marked depletion of T_EM _cells (Figures 5b and 5c). At day 21, a significant increase in the number of intestinal CD4+CCR5- T_N _cells compared with naive controls was observed (30.9% ± 9.3%, p = 0.02; Figures 5a and 5c). Concurrently, restoration of a clearly distinguishable population of CD4+CD95+CD28- T_EM _cells was observed (Figure 5c). At day 125 elevated numbers of intestinal T_N _remained (24.9% ± 3.1%, p = 0.06), but the proportion of CD4+CCR5+ T cells was now significantly increased, compared to naive controls, (47.46% ± 5.51%, p < 0.05) and the CD4+ T_EM _subset was further restored by mostly CCR5+ cells (Figures 5b and 5c).

### Attenuated SIV differentially modulates intestinal LPL and IEL CD4+ T cells

**Figure 6:**
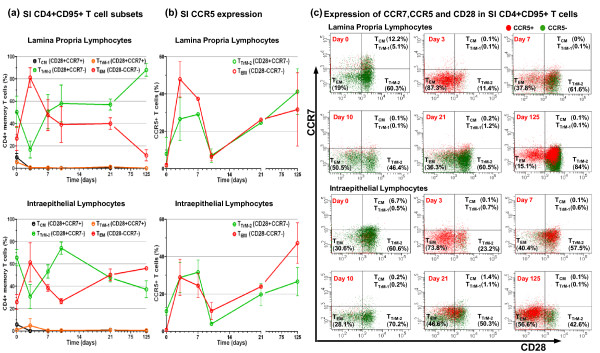
**Vaccination with attenuated SIV differentially modulates LPL and IEL CD4+CCR5+ T cell memory subsets**. Following vaccination of cynomolgus macaques (n = 20) with live attenuated SIV the percentages of LPL and IEL CD4+CCR5+ T_CM_, T_TrM-1_, T_TrM-2 _and T_EM _cells from the SI was determined at days 0, 3, 7, 10, 21 and 125 post inoculation. Transient increases in the percentage of SI LPL and IEL CD4+ T_EM _cells with a concomitant decrease in CD4+ T_TrM-2 _cells was observed at day 3 **(a)**. Increased percentages of SI LPL and IEL CD4+CCR5+ T_TrM-2 _and T_EM _cells was observed at days 3 and 7 **(b)**. Immunostaining of small intestine LPL and IEL gated on CD3+CD4+CD95+ memory T cells from one representative animal from each time point are shown **(c)**. In each panel the upper right hand quadrant shows CD28+CCR7+ T_CM _and T_TrM-1 _cells, the lower right quadrant CD28+CCR7- T_TrM-2 _cells and the lower left quadrant CD28-CCR7- T_EM _cells. CD4+ T_TrM-1 _cells are essentially CCR5+ T_CM _cells. Percentages shown give the proportion present in each of these subsets. CD4+CD95+CCR5+ T cells are shown in red and CD4+CD95+CCR5- T cells in green. T_CM_: central memory, T_TrM-1_: transitional memory subset-1, T_TrM-2_: transitional memory subset-2, T_EM_: effector memory, IEL: intraepithelial lymphocytes, LPL: lamina propria lymphocytes, SI: small intestine.

Using the differentiation sequence defined for rhesus macaque CD4+ memory T cells where CCR7 and then CD28 are sequentially down regulated [58,59], it is possible to subdivide cynomolgus macaques CD4+ memory T cells into 4 subsets, CD28+CCR7+ T_CM _→ CD28+CCR7+ T_TrM-1 _→ CD28+CCR7- T_TrM-2 _→ CD28-CCR7- T_EM_, where the transitional memory subset-1 (T_TrM-1_) are essentially CCR5+ T_CM _cells. Using this regimen, we investigated further CCR5 expression by intestinal LPL and IEL CD4+ memory T cells following vaccination with live attenuated SIV. Rather than T_CM _cells, defined previously as CD28+ cells, we found that the majority of intestinal CD4+ memory T cells in naive cynomolgus macaques were in fact of the transitional memory subset-2 (T_TrM-2_) and negative for CCR5 expression (Figures 6a, b and 6c). Representative dot plots showing CCR5 staining of SI LPL and IEL CD4+ memory subsets are shown in Figure 6c. Modest frequencies of CCR5+ cells were mostly found in the T_TrM-1 _subset of LPL and the T_TrM-2 _subset of IEL of naive macaques (Figures 6b and 6c). At day 3, there was a dramatic increase in CCR5 expression by both CD4+ T_EM _and T_TrM-2 _cells accompanied by a large population shift to a T_EM _cell phenotype, in both LPL and IEL (Figures 6b and 6c). At the same time CD4+ T_CM _and T_TrM-1 _cells were depleted within LPL and IEL populations (Figures 6a and 6c). At day 7, the majority of LPL and IEL CD4+ T_EM _cells appeared to have either reverted to a T_TrM-2 _cell phenotype or were depleted, whilst the remaining CD4+ T_TrM-2 _cells were largely positive for CCR5 expression (Figures 6b and 6c). Very few LPL and IEL CD4+ T_CM _and T_TrM-1 _cells were detected at day 7 and at all time points investigated thereafter (Figures 6a and 6c). At day 10, CCR5 expression within the CD4+ T_EM _and T_TrM-2 _subsets, within LPL and IEL, was almost completely lost (Figures 6b and 6c). The remaining LPL and IEL CD4+ T_EM _and T_TrM-2 _cells were largely negative for CCR5 expression (Figures 6b and 6c). At day 21, a higher frequency of IEL CD4+CCR5+ T_EM _cells was observed, but no marked increase was seen in the frequency of LPL CD4+CCR5+ T cells (Figures 6b and 6c). At day 125, the proportion of IEL CD4+CCR5+ T_EM _cells increased further although T_TrM-2 _cells were largely CCR5- (Figures 6b and 6c). By contrast, the frequency of LPL CD4+ T_EM _cells was greatly reduced and T_TrM-2 _cells increased at day 125 (Figure 6a). Marked increases in the frequency of LPL CD4+CCR5+ cells at day 125 were mostly confined to the T_TrM-2 _subset (Figures 6b and 6c), further distinguishing it from the IEL compartment at this time.

## Discussion

Live attenuated SIV vaccines provide potent protection, but the detailed properties of this protection appear to vary depending upon the model system studied. In this report, we describe further studies to characterise the mechanism of protection conferred by a minimally *nef*-deleted attenuated vaccine derived from SIVmac251, called SIVmacC8 [61], in (Mauritian derived) cynomolgus macaques. Vaccination of cynomolgus macaques with SIVmacC8 protects against infection with virus infected cells as well as cell free virus [5], develop by 3 weeks [43] and paradoxically protects against a genetically heterologous virus challenge better (N. Berry personal communication) than a highly vigorous homologous virus challenge [37]. Since we have been unable to identify a mechanism of protection amongst adaptive immune responses that develop following vaccination, either by passive transfer of immune serum [24] or CD8+ T cell depletion [34,62], we investigated whether other responses to vaccination may contribute to protection. It is accepted that infection with wild-type SIV rapidly induces a depletion of CD4+CCR5+ memory T cells in the GALT [54-57]. Therefore, we speculated whether a similar effect following vaccination with SIVmacC8 would result in target cell depletion, preventing subsequent virus challenges from infecting the GALT and so preventing a systemic infection from being established. The data indicate that, following inoculation of SIVmacC8, marked dynamic changes in CD4+ T cell populations occur that may not only contribute to the protective effect of vaccination, but could also be instrumental in regulating the kinetics of replication by this virus.

Previous reports of T cell dynamics in the GALT of rhesus macaques, following infection with attenuated SIV, suggested that minimal changes occurred since the total CD4+population remained unaltered [48,58]. This also appeared to be the case for cynomolgus macaques vaccinated with SIVmacC8. However, more detailed analyses of CD4+CCR5+ memory T cell populations revealed a more dynamic picture of events.

By immunostaining with antibodies to CD3, CD4, CCR5, CD28, CD95 and CCR7 markers, it was possible to define the naive and memory helper T cell compartments in considerable detail. Prior to vaccination with SIVmacC8, the low level of CCR5 expression by CD4+ T_EM _cells in naive cynomolgus macaques would be anticipated with a lack of activation and proinflammatory Th1 responses [63-65]. By contrast, within 3 days of vaccination when the primary viraemia is first detectable, a dramatic expansion of intestinal CD4+CCR5+ T_EM _cells was detected, consistent with an acute Th1 proinflammatory response [66-68]. This expansion of intestinal CD4+CCR5+ T_EM _cells was probably a result of activation-associated upregulation of CCR5 expression by CD4+CCR5- T cells, since concurrent reductions in less differentiated CD4+ T_CM_, T_TrM-1 _and T_TrM-2 _cells were detected. Alternative explanations such as the proliferation of intestinal CD4+CCR5+ T_EM _cells or an influx of CD4+CCR5+ T_EM _cells into the intestinal mucosa are less likely because of the limited proliferative potential of T_EM _cells [69] or the need for co-ordinated outflow of CD4+CCR5- T cells to balance overall CD4+ T cell percentages.

The marked expansion in the activated intestinal CD4+CCR5+ cell population in the absence of acquired immune responses would provide large numbers of target cells in which SIVmacC8 could replicate readily. Indeed, virus infected cells are detectable in the small intestine from day 3 by immunohistochemistry (D. Ferguson personal communication). However, it is not known whether this series of events reflects SIV exploiting a generic host response to infection or whether it is a result of specific viral factors driving events. Nevertheless, not only did the early expansion of intestinal CD4+CCR5+ T cells, detectable from day 3, appear to "fuel" the increases in plasma SIV RNA loads at days 3 and 7, but also the loss of LPL and IEL CD4+CCR5+ T_EM _cells from day 7 also augured the end of the primary viraemia from day 10 when there were dramatic reductions in remaining LPL and IEL CD4+CCR5+ T_TrM-2 _cells to pre-infection levels. These data, which were virtually indistinguishable from those previously reported for this virus [70], suggest that the kinetics of SIVmacC8 primary viraemia may be regulated by the availability of target cells as much as the development of anti-viral immune responses. Studies of the infection of Chinese rhesus macaques with pathogenic SIV have also reported that peak plasma SIV RNA loads were associated with the loss of intestinal CD4+CCR5+ T cells [71,72]. Nevertheless there is considerable evidence that acquired anti-SIV immune responses, such as CD8+ cytotoxic T cells, regulate viral loads [33,34,73-76]. Intriguingly, in a previous report of the primary viraemia of SIVmacC8 during profound CD8+ cell depletion, whilst the peak SIV RNA loads were approximately 300 times higher in the absence of CD8+ T cells, SIV RNA loads declined prior to recovery of detectable CD8+ T cells [34], indicating that other mechanisms must also contribute to the control of the primary viraemia.

The dramatic loss of intestinal CD4+CCR5+ T cells between days 7 and 10, post vaccination with attenuated SIV, may be due to indirect mechanisms such as CD95 dependant apoptosis [56] as well as direct lytic viral replication. However, it is unclear why overall CD4+ T cell percentages were not then reduced, as per wild-type SIV infection [48,58]. It may be that lower cell-associated virus loads and the non-pathogenic nature of attenuated SIV infection reduce rates of CD4+CCR5+ T cell attrition thereby allowing T cell homeostatic repopulation of the GALT to be sustained. Such repopulation could originate from outside the GALT or through expansion of intestinal CD4+CCR5- T_EM _and T_TrM-2 _cell populations. Though at this time there were few naive T_N _cells detectable to suggest repopulation, and T_EM _cells have been reported to have limited proliferative capacity [77,78]; however, the high proliferative capacity of CD4+ T_TrM-2 _[59] cells could have been able to support that repopulation. Alternatively, down regulation of CCR5 expression on CD4+CCR5+ T cells may better account for the dynamic changes observed between days 7 and 10, that is not necessarily due to depletion. In order to address this possibility we need to investigate whether CD4+CCR5- cells harbour attenuated SIV, as others have found with pathogenic SIV [57].

Nevertheless, at days 21 and 125, there was a dramatic increase in intestinal T_N _cells that may signify an influx of repopulating cells to replace ongoing losses and maintain homeostasis, as has been reported following infection with pathogenic SIV [48]. Since the second dramatic increase in CD4+CCR5+ intestinal T cells at day 125 occurred without the appearance of increased SIV RNA loads, other factors not present during the acute infection must be restricting SIV replication, preventing further loss of intestinal CD4+CCR5+ T cells. Further work is needed to determine whether adaptive immune or other anti-viral responses, such as retroviral superinfection resistance, are involved at these later times specifically [79]. Whatever the mechanism identified, it needs to be able to account for the characterised properties of protection conferred by live attenuated vaccines in the species of macaque being studied.

One of the difficulties for understanding vaccine protection conferred by live attenuated SIV has been the frequently confusing, if not conflicting data, obtained by different groups using related vaccine models but in different species of macaque. In this report, we found a much lower frequencies of intestinal CD4+CCR5+ T cells (16.16% ± 2.44%) in naive cynomolgus macaques compared with Indian rhesus macaques, where the level of CCR5 expression by CD4+ intestinal T cells is reported to be >60% [57]. Intriguingly, average levels of CCR5 expression on CD4+ intestinal T cells of SIV natural hosts is reported to be considerably lower those we have described. For example, 9.13% for African green monkeys and 1.2% for sooty mangabeys [80]. It may be hypothesised that this lower level of CCR5 expression by CD4+ T cells may contribute to the more limited damage of the immune system caused by wild-type SIV in these hosts as there would be reduced numbers of target cells susceptible to infection and destruction at any time [80]. Conversely, the higher levels of CCR5 expression on peripheral CD4+ T cells of Indian versus Chinese rhesus macaques, 21.8% ± 7.7% and 6.7% ± 4.6% respectively, could contribute to the sustained high virus load and faster disease progression seen in Indian rhesus macaques infected with SIV [72,81]. If alterations in the frequency of these same CD4+ T cell subsets contribute not only to viral kinetics but also to the protection mediated by live attenuated SIV, then it may be anticipated that undertaking a similar vaccine study in macaques of different species could result in distinct outcomes.

## Conclusions

Vaccination with live attenuated SIV causes dynamic changes and chronic expansion of CD4+CCR5+ intestinal T cell memory subsets, more consistent with immune activation than target cell depletion. The profile of high frequencies of CD4+CCR5+ T cells detectable in the GALT after vaccination is not identical to those found in naive animals or expanded during the early stages of the primary viraemia, implying lasting immune modulation. Understanding the impact of the immune modulation caused by attenuated SIV and the mechanism(s) involved may provide insight into the development of novel vaccine approaches or therapies that safely reproduce this protection.

## Methods

### Experimental Outline

In this study, twenty D-type-retrovirus-free juvenile cynomolgus macaques (Macaca fascicularis), housed and maintained in accordance with UK Home Office guidelines for the care and maintenance of nonhuman primates, were used. Animals were sedated with ketamine hydrochloride before inoculation of virus or venepuncture and killed humanely by an overdose of anaesthetic. The vaccine virus SIVmacC8 is a clone of a rhesus passage of wild-type SIVmac251 [8] attenuated by a 12 bp in-frame deletion in the *nef *open reading frame and two further conservative amino acid changes [61]. Cynomolgus macaques were inoculated with vaccine virus by intravenous injection of 5000 TCID_50 _of the 9/90 pool of SIVmacC8 [61], which has an end-point titre of 10^4 ^TCID_50_/ml on C8166 cells, and were sacrificed in pairs on days 3, 7, 10, 21 and 125 (n = 2) in the first study and days 3, 10 and 21 (n = 2) in a second study for analysis and comparison with naive macaques (n = 4) of CCR5 expression across CD4+ T cell memory subsets.

### Tissue collection

PBMCs were isolated by density gradient centrifugation as previously described [34]. Spleen, PLN and MLN cells were isolated by mechanical tissue disaggregation (Medimachine, BD Biosciences, Oxford, UK). LPL and IEL were isolated from the SI and LI. Briefly, intestinal sections were opened longitudinally, cut into 5 cm segments and washed with cold HBSS (Gibco^®^Invitrogen Ltd., Paisley, UK). Segments were then incubated in cold Ca^2+ ^and Mg^2+ ^free HBSS (Gibco^®^Invitrogen Ltd., Paisley, UK) containing 10 mM Dithiothreitol (Sigma-Aldrich, Dorset, UK) on an orbital shaker for 45-60 minutes at 4°C. After incubation IEL were collected as the filtrate from a 100 μm cell strainer (BD Biosciences, Oxford, UK). Remaining intestinal tissue was then incubated with warm collagenase solution (0.5 mg/ml) on an orbital shaker at 37°C for 30-45 minutes. After incubation LPL were collected as the filtrate from a 100 μm cell strainer (BD Biosciences, Oxford, UK). All extracted filtrates were centrifuged at 400 g for 10 min, pellets re-suspended in RPMI 1640 (Sigma-Aldrich, Dorset, UK), layered onto FCS and spun at 400 g for 10 min. Cell pellets were re-suspended in RPMI 1640 containing 2 mg/ml DNAse (Sigma-Aldrich, Dorset, UK) and incubated for 20 min on shaker at 37°C. Cell suspensions were layered over a 35% Percoll gradient (Sigma-Aldrich, Dorset, UK) which was layered over a 65% Percoll gradient and centrifuged at 500 g for 30 min. Lymphocytes present at the interface between the 35% and 65% Percoll layers were aspirated and cells washed twice in RPMI 1640. Prior to staining cells were further processed using a "Dead Cell Removal Kit", according to manufacturer's instructions, to reduce debris (Miltenyi Biotec, Surrey, UK).

### Detection and quantification of SIV RNA, DNA and cell-associated virus

SIV RNA levels in plasma were determined by quantitative real-time RT-PCR (qRT-PCR) as previously described [37]. Viral RNA was extracted from 140 μl plasma using viral RNA mini-kits (QIAamp; Qiagen, Crawley, UK) then eluted in a total volume of 50 μl AVE buffer. RNA (5 μl) extracted from reference or experimental samples were amplified in triplicate using the Brilliant QRT-PCR plus Core Reagents one-step kit (Agilent Technologies Inc., CA, USA). Oligonucleotide primers and probe sequences, located in conserved regions of gag, were optimized at 300 and 100 nM respectively [37]. A value of 1.3 log10 SIV RNA copies per ml is below the cut-off for quantification in this assay. Cell-associated virus loads of isolated lymphoid cells were determined by co-culture with C8166 cells, and the presence of replicating virus was confirmed by syncytia identification or by antigen capture at 28 days [62].

Genomic DNA was extracted from 10^6 ^purified intestinal lymphocytes (as described above) and proviral SIV gag DNA levels determined by quantitative PCR (qPCR), using the same primer/probe sequences as the qRT-PCR assay [37]. The concentration added to each PCR assay was determined retrospectively using a fluorometic DNA quantification kit (Sigma-Aldrich, Dorset, UK) in a microtitre format. Aliquots of DNA (1 μl) were assayed in triplicate using a Taqman Universal PCR Master Mix (ABI) against a standard curve of the p2-LTR plasmid [70] serially diluted in herring sperm DNA [37]. SIV DNA levels were expressed as copies of SIV DNA per 10^5 ^mononuclear cells (MNC) with an absolute limit of detection being 1 SIV DNA copy per 10^5 ^cells.

### Analysis of CCR5 expression by T cell memory subsets

Expression of CCR5 within T cell subsets defined by CD3-FITC (clone FN18, AbD Serotec, UK), CD4-APC-Cy7 (clone OKT4, Biolegend, Cambridge, UK), CD8-AmCyan (clone SK1, BD Biosciences, Oxford, UK), CD28-PerCP-Cy5.5 (clone CD28.2, eBioscience Ltd., Hatfield, UK), CD95-PE-Cy7 (clone DX2, eBioscience Ltd., Hatfield, UK) and CCR7-FITC (clone 150503, R&D systems, Abingdon, UK) was assessed by flow cytometry. Within the CD3+CD4+ (helper) T cell subset, naive (CD95-CD28+, T_N_), central memory (CD95+CD28+ or CD95+CD28+CCR7+CCR5-, T_CM_), transitional memory subset-1 (CD95+CD28+CCR7+CCR5+, T_TrM-1_), transitional memory subset-2 (CD95+CD28+CCR7-, T_TrM-2_) and effector memory (CD95+CD28- or CD95+CD28-CCR7-, T_EM_) distinctions were made [59,60]. Staining was performed as previously described [29]. Acquisition was performed using a BD FACSCanto II and analysed using BD FACSDiva software (BD Biosciences, Oxford, UK). At least 10,000 CD3+CD4+ events were collected for subset analysis.

Immunohistochemical analysis was performed as previously described [82]. Briefly formaldehyde fixed, paraffin embedded tissue sections were de-waxed, and re-hydrated before being incubated with 50 μg/ml proteinase K (Roche Products Ltd., Welwyn Garden City, UK) in PBS pH7.4 for 15 minutes at 37°C to unmask target antigens followed by immuno-labelling with anti-CCR5 (3A9, BD Biosciences, Oxford, UK). Bound antibodies were visualized using the Vector ABC amplification system (Vector Laboratories, Peterborough, UK) in combination with a biotinylated universal anti-mouse/rabbit secondary antibody (Vector Laboratories, Peterborough, UK).

### Statistical analysis

A Kruskal-Wallis test followed by Dunn's post test was used for comparison of CD3+CD4+ and CD3+CD4+CCR5+ counts and plasma SIV vRNA loads at each time point measured. Values expressed are mean ± standard error of means (SEM). All reported P values were two sided at the 0.05 significance level determined using Prism 5 software (Graph Pad Software, CA, USA).

## Competing interests

The authors declare that they have no competing interests.

## Authors' contributions

RS, NA and NB conceived and designed the experiments; BL, CH, DF, DS, JH, MP, RQ, MR and WE performed the experiments; RS and BL analysed the data; RS, BL and NA wrote the paper. All authors read and approved the final manuscript.
